# Amantadine in the Treatment of Pathological Gambling: A Case Report

**DOI:** 10.3389/fpsyt.2012.00102

**Published:** 2012-11-27

**Authors:** Mauro Pettorruso, Giovanni Martinotti, Marco Di Nicola, Marco Onofrj, Massimo Di Giannantonio, Gianluigi Conte, Luigi Janiri

**Affiliations:** ^1^Department of Psychiatry, Drug Addiction Unit, Catholic University Medical SchoolRome, Italy; ^2^Department of Neuroscience and Imaging, University G. d’AnnunzioChieti, Italy; ^3^Department of Neurology, University G. d’AnnunzioChieti and Pescara, Italy

**Keywords:** pathological gambling, amantadine, addiction, glutamate, dopamine, treatment, glutamatergic drugs

## Abstract

Despite almost a decade of intense research, effective treatment strategies for Pathological Gambling (PG) remain very challenging. This paper details a case report suggesting that the treatment of PG may benefit from the use of the non-specific glutamate blocker amantadine. The drug was well-tolerated and effective, leading to a 43–64% reduction in severity of gambling symptoms (as measured with G-SAS). Our result is discussed in the context of the glutamatergic hypothesis of addiction and in light of previous observations on the potential impact of glutamatergic agents in the treatment of PG. The role of the dopaminergic system, and its interaction with the glutamatergic system, is also explored. Further studies are required to define the true benefits of amantadine for the treatment of PG.

## Background

Pathological gambling (PG) is characterized by persistent and maladaptive gambling behavior, whereby individuals engage in frequent and repeated episodes of gambling despite serious adverse consequences (Hodgins et al., 2011). Gambling disorder affects 0.2–5.3% of adults worldwide; the devastating consequences of this behavioral disturbance often entail severe damage to the lives of patients and their families. To date, there is no FDA-approved treatment for PG, despite almost a decade of intense research, and effective treatment strategies remain very challenging.

Recently, it has been proposed that PG be included in the diagnostic category of Substance Use and Addictive Disorders of the next edition of the Diagnostic and Statistical Manual of Mental Disorders (DSM-V; Petry, 2010). The clinical and biological similarities between PG and drug addiction (Potenza, 2008) suggest that PG patients may benefit from medication used to treat drug addiction and that pathophysiological models for drug addiction may be relevant to PG as well. It has been recently proposed that addiction be viewed as the result of an impaired ability to inhibit drug seeking in response to environmental contingencies, due to alterations in glutamate (Glu) homeostasis, with combined activation of sensitized dopamine (DA) and *N*-methyl-d-aspartate (NMDA) glutamatergic receptors (Kalivas, 2009).

Pathological Gambling has been presumed to be modulated mainly by brain DA and Glu, though findings are contrasting. DA is implicated in rewarding, reinforcing, and addictive behaviors. In drug addiction, data support the existence of a hypodopaminergic state at both the presynaptic and postsynaptic levels (Melis et al., 2005). Alterations in dopaminergic pathways may underlie the seeking of rewards (i.e., gambling) that trigger DA release and produce feelings of pleasure. While the acute rewarding effects of addictive behaviors seem to be mediated by enhanced DA transmission and DA release may reinforce reward learning, Glu may be implicated in long-lasting neuroadaptations in the corticostriatal circuitry, that represents the putative neural substrate of enduring vulnerability to relapse (Kalivas, 2009). The imbalance in Glu homeostasis engenders changes in neuroplasticity that impair communication between the prefrontal cortex and the nucleus accumbens, thus favoring engagement in reward-seeking behaviors, such as PG (Kalivas and Volkow, 2011).

Amantadine is an antiglutamatergic drug with NMDA receptor antagonistic properties. Amantadine also has additional actions on dopaminergic neurotransmission; it increases DA release, has direct effects on DA receptors, and inhibits DA reuptake (Stromberg et al., 1970). Amantadine was originally approved as an antiviral agent (Davies et al., 1964) as it inhibits influenza A virus replication. With its pro-dopaminergic actions, amantadine was later approved for Parkinson’s disease (PD) and for drug-induced parkinsonism (Schwab et al., 1969). It continues to be a widely used and well-tolerated drug in the treatment of parkinsonian movement disorders and may also slow the progression of PD (Blanchet et al., 2003), ameliorate chronic pain (Fisher et al., 2000), and improve recovery from traumatic brain injury (Meythaler et al., 2002). Pro-dopaminergic actions and NMDA antagonistic actions are suggestive of amantadine’s possible efficacy for depression. The adverse effects of amantadine are generally mild. The most commonly reported side effects include nausea, dizziness, and insomnia. Confusion, ataxia, headache, agitation, and fatigue have been reported, but are less frequent. Psychiatric side effects include depression, anxiety, irritability, and dream abnormality. Psychosis, euphoria, and amnesia have also been reported. Suicidal ideation, aggressive behavior, delirium, delusions, paranoid reactions, and impulse control symptoms have been reported, but are rare.

Based on amantadine’s properties of stimulating DA release, several studies on its use in cocaine addiction have been conducted, though findings are contrasting. In cocaine-dependent patients with severe withdrawal symptoms, Kampman et al. (2000) found that amantadine was effective. Two double-blind trials yielded similar results (Alterman et al., 1992; Shoptaw et al., 2002). However, several other placebo-controlled trials have not confirmed amantadine’s effectiveness in cocaine dependence (Giannini et al., 1989; Weddington et al., 1991; Kampman et al., 2006). Amantadine has also been reported to be effective in reducing PG in patients with PD (Thomas et al., 2010). In the latter study, amantadine resolved PG in seven patients after 4 days of treatment; conversely, amantadine withdrawal may have been associated with the reappearance of the disorder. Although not previously studied in PG, we hypothesized that, on the basis of these findings, and on its potential to interact with Glu homeostasis and DA function, amantadine would reduce gambling craving as well as gambling behavior. In this report, we present the first experience using amantadine to treat PG.

## Case Presentation

The present case concerned a 47-year-old married male, unemployed, previously diagnosed with PG (about 10 years ago). He first started engaging in problematic gambling at horse betting and slot machines 25 years ago. Though he has no criminal history, he has run up about 20,000 euros in debts due to PG. The patient also reported a history of opioid dependence, in remission for 12 years.

At intake assessment, the patient met DSM-IV-TR (American Psychiatric Association, 2000) and ICD-10 criteria for PG, and was currently experiencing a Major Depressive Episode (MDE) according to DSM-IV-TR. He also met criteria for alcohol dependence, nicotine dependence, and occasional cocaine abuse. The patient did not receive psychological or pharmacological treatments for the opioid and alcohol dependence. Personality traits were explored using the Temperament and Character Inventory – Revised (TCI-R; Cloninger et al., 1993; Martinotti et al., 2008): he reported low scores on Harm Avoidance, Persistency, Self-Transcendence, and high scores on Cooperativeness. Impulsivity was measured with the Barratt Impulsiveness Scale, Version 11 (BIS-11; Patton et al., 1995), and a score of 73 was obtained, indicating high levels of impulsivity. The Hamilton Depression Rating Scale (HAM-D, Hamilton, 1960) and the Young Mania Rating Scale (YMRS; Young et al., 1978) were also administered in order to assess mood symptoms; a score of 17 was obtained on the HAM-D and a score of 2 was obtained on the YMRS. Gambling craving was assessed with the Gambling Symptom Assessment Scale (G-SAS; Kim et al., 2009) and a score of 44 was obtained, which reflects extreme severity of the gambling urge. The patient was initially assigned to a semi-residential treatment program at the Day Hospital of Psychiatry of the Catholic University in Rome, during which he received antidepressant medication (escitalopram 20 mg/day) and joined rehabilitation groups (two sessions per week) specifically aimed at managing PG and raising illness awareness. Alcohol withdrawal symptoms were treated with pregabalin (225 mg/day) and sleep disturbance with trazodone (50 mg/day).

**Figure 1:**
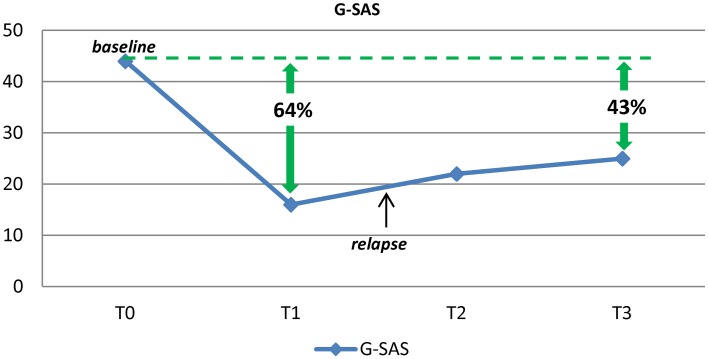
**Results of Gambling Symptoms Assessment Scale (G-SAS), at baseline and follow up**.

After 4 weeks of treatment, though the depressive condition had improved significantly (total score HAM-D = 2), he continued to gamble and no change was reported in gambling craving scores, as measured with the G-SAS. Three months after the first assessment, he was referred to the psychiatric ward of the clinic “Villa Maria Pia” in Rome. Upon admission, he did not present acute psychopathological conditions, with the exception of alcohol and gambling craving. Treatment with amantadine was initiated, after the patient gave his written informed consent. The study consisted of 10 weeks of open-label amantadine. Amantadine was administered with a titration schedule of 50 mg daily for 1 week, 50 mg twice daily (bid) for 2 weeks, and 100 mg + 50 mg for the rest of the study period. During all the study period, the patient continued to receive the previous treatment (pregabalin 225 mg/day; trazodone 50 mg/day) to treat alcohol withdrawal symptoms (Di Nicola et al., 2010). At baseline, G-SAS was administered and the subject reported the previous score of 44. Depressive and manic/hypomanic symptoms were assessed using the HAM-D and the YMRS (see Table 1). HAM-D, YMRS, and G-SAS were repeated after 2, 4, and 8 weeks. After the first 2 weeks of treatment the patient was discharged from the clinic and continued receiving amantadine as an outpatient at the Day Hospital of Psychiatry of the Catholic University in Rome. Safety assessment at each visit included evaluation of sitting blood pressure, heart rate, and weight. The patient tolerated the medication well, without any side effects. ECG results were normal throughout the entire study period, and no effect on QTc interval was observed. Also, the patient did not report any significant anticholinergic side effects. During the study period, the patient reported a reduction of 43–64% in G-SAS scores (with a reduction from extreme to mild-moderate gambling urge; Figure 1), no change in mood assessment was observed, and only one relapse in PG behaviors was reported.

**Table 1:** **Changes in study measures of the patient with pathological gambling treated with amantadine**.

	Baseline	2 weeks*	4 weeks*	8 weeks*
G-SAS	44	16	22	25
HAM-D	2	3	6	5
YMRS	5	3	4	3
Gambling relapse (n/days)	18/30	0/14	1/14	0/30
Alcohol TLFB	5/30	0/14	2/14	3/30

G-SAS, Gambling Symptom Assessment Scales; HAM-D, Hamilton Depression Rating Scale; TLFB, Alcohol Timeline Follow Back; YMRS, Young Mania Rating Scale. *After administration of amantadine.

## Discussion

To our knowledge, this is the first case report examining the efficacy of amantadine in treating PG patients, without comorbid PD. We found that PG symptoms improved significantly during the study period. This suggests that simultaneous pharmacological modulation of the glutamatergic and dopaminergic systems may reduce gambling in PG.

Amantadine is an old drug, but new potential uses were only recently discovered (Rajput et al., 1998; Hubsher et al., 2012). Besides its established use in the treatment of levodopa-induced dyskinesia (Pappa et al., 2010; Sawada et al., 2010), amantadine has also recently been studied in PD patients as an additional treatment option for punding (a stereotyped behavior characterized by intense fascination with complex, excessive, non-goal-oriented, repetitive activities; Fasano et al., 2011) and PG (Thomas et al., 2010), in light of the hypothesis that both dyskinesias and compulsive behaviors (such as PG and punding) represent part of a pathological continuum secondary to abnormal dopaminergic stimulation in the basal ganglia (Voon et al., 2009).

There is substantial evidence indicating that pharmacological treatments targeting glutamatergic transmission are of potential utility in the treatment of drug addiction. Given that neurobiological findings indicate that PG and drug addiction share common etiopathological pathways (Potenza, 2008), drugs targeting glutamatergic transmission could be of use for the treatment of behavioral addictions (i.e., PG) as well (Kalivas, 2009; Olive et al., 2012). Previous glutamatergic treatment strategies for PG proved to reduce gambling urges and behavior. A small clinical trial showed that *N*-acetylcysteine, an amino acid that seems to restore extracellular Glu concentration in the nucleus accumbens, lowered scores on the Yale Brown Obsessive Compulsive Scale adapted for PG (PG-YBOCS; Grant et al., 2007). In line with this finding, a subsequent open-label study reported that memantine, a non-competitive antagonist at the NMDA receptor, decreased PG-YBOCS scores and time spent gambling (Grant et al., 2010), also improving neurocognitive function related to cognitive flexibility.

The dopaminergic system has also been targeted, for the treatment of both drug and behavioral addictions (Diana, 2011). Neuroimaging research suggests that the dopaminergic mesolimbic pathway from the ventral tegmental area to the nucleus accumbens might be involved in PG. Pathological gamblers have diminished ventral striatum, ventromedial prefrontal cortex, and ventrolateral prefrontal cortex activity during rewarding events, suggesting that there is a blunted neurophysiological response to rewards and losses (Reuter et al., 2005; de Ruiter et al., 2009). To date, treatment strategies in PG patients based on dopaminergic system manipulation have proven to be ineffective. Bupropion, a DA and noradrenaline reuptake inhibitor, showed no benefit over placebo (Black et al., 2007). Antagonists at dopamine D2/D3 receptors enhanced gambling-related motivation and behaviors in PG patients (Zack and Poulos, 2007). In pathological gamblers, multiple investigations have observed alterations in frontal regions and in the ventral striatum (nucleus accumbens), a brain region with dopaminergic innervation widely implicated in reward processing (Everitt and Robbins, 2005). It has been hypothesized that two mechanisms of midbrain and striatal dopaminergic projections may be involved in PG: hypersensitivity to reward and sustained activation toward uncertainty (Linnet et al., 2012). Furthermore, Joutsa et al. (2012) suggested that the dopaminergic response to reward-predicting stimuli and the link between addiction severity and DA release in pathological gamblers may be relevant to the onset and course of PG. As an indirect DA agonist, amantadine may be able to stimulate the release of DA and potentially restore the physiological activity of the DA system, yielding significant clinical improvements in PG patients (reduction of craving and relapse).

Amantadine may act as a regulator of the complex interactions between the glutamatergic and dopaminergic systems, acting simultaneously on both systems, in ways that need to be better explored. Takahashi et al. (1996) proposed that amantadine may stimulate D2 receptors distributed in Glu nerve terminals, thereby enhancing Glu release in the striatum. Enhanced Glu transmission, in turn, would induce a further increase in DA through NMDA receptors located in DA nerve terminals. Intriguing hypotheses can be formulated in light of the recent discovery of the existence of a co-release of Glu and DA in reward-related areas. Based on the finding of VGLUT2 expression in DA neurons of brain regions projecting to the nucleus accumbens and on electrophysiological recordings showing glutamatergic transmission in mesoaccumbal slice preparations (Chuhma et al., 2004), it has been suggested that VGLUT2-mediated Glu co-transmission might have a role in reward-relevant pathways (El Mestikawy et al., 2011). Further studies are needed to better understand the action of amantadine on neural systems involved in PG.

## Concluding Remarks

Our data seem to confirm the utility of targeting the Glu system for the treatment of PG, and suggest that the non-specific Glu blocker amantadine may possibly be a viable treatment option. Since amantadine presumably modulates Glu homeostasis, we hypothesize that its therapeutic action is linked to the restoration of prefrontal projections to the ventral striatum (nucleus accumbens), thus reversing neuroplasticity-based pathological changes putatively determined by addictive behaviors (Kalivas, 2009).

Though the use of amantadine in the treatment of PG appears to be promising, further studies are certainly needed. Considering that this is the only case report describing such a new therapeutic use for amantadine, the generalizability of the efficacy results is limited. Future investigations would benefit from placebo-controlled clinical trials to further outline the true benefits of amantadine for the treatment of PG. Future studies should also focus on the functional connections between dopaminergic and glutamatergic systems, in order to shed light upon the complex neurobiological mechanisms underlying the development of maladaptive gambling behavior.

## Conflict of Interest Statement

The authors declare that the research was conducted in the absence of any commercial or financial relationships that could be construed as a potential conflict of interest.
